# Prediction of Surface Subsidence in Mining Areas Based on Ascending-Descending Orbits Small Baseline Subset InSAR and Neural Network Optimization Models

**DOI:** 10.3390/s24154770

**Published:** 2024-07-23

**Authors:** Kangtai Chang, Zhifang Zhao, Dingyi Zhou, Zhuyu Tian, Chang Wang

**Affiliations:** 1 Institute of International Rivers and Eco-Security, Yunnan University, Kunming 650500, China; ckt975907182@163.com (K.C.); zhoudingyi@mail.ynu.edu.cn (D.Z.); 2 School of Earth Sciences, Yunnan University, Kunming 650500, China; 3 Yunnan International Joint Laboratory of China-Laos-Bangladesh-Myanmar Natural Resources Remote Sensing Monitoring, Kunming 650500, China; 4 Research Center of Domestic High-Resolution Satellite Remote Sensing Geological Engineering, Kunming 650500, China; 5 Yunnan Key Laboratory of Sanjiang Metallogeny and Resources Exploration and Utilization, Kunming 650051, China; 6 College of Geological Engineering and Geomatics, Chang’an University, Xi’an 710054, China; t157007102@outlook.com; 7 School of Information Engineering, Chang’an University, Xi’an 710064, China; wangchang2437@163.com

**Keywords:** Sentinel-1A, SBAS-InSAR, two-dimensional deformation decomposition, subsidence prediction, optimization algorithm

## Abstract

Surface subsidence hazards in mining areas are common geological disasters involving issues such as vegetation degradation and ground collapse during the mining process, which also raise safety concerns. To address the accuracy issues of traditional prediction models and study methods for predicting subsidence in open-pit mining areas, this study first employed 91 scenes of Sentinel-1A ascending and descending orbits images to monitor long-term deformations of a phosphate mine in Anning City, Yunnan Province, southwestern China. It obtained annual average subsidence rates and cumulative surface deformation values for the study area. Subsequently, a two-dimensional deformation decomposition was conducted using a time-series registration interpolation method to determine the distribution of vertical and east–west deformations. Finally, three prediction models were employed: Back Propagation Neural Network (BPNN), BPNN optimized by Genetic Algorithm (GA-BP), and BPNN optimized by Artificial Bee Colony Algorithm (ABC-BP). These models were used to forecast six selected time series points. The results indicate that the BPNN model had Mean Absolute Errors (MAE) and Root Mean Squared Errors (RMSE) within 7.6 mm, while the GA-BP model errors were within 3.5 mm, and the ABC-BP model errors were within 3.7 mm. Both optimized models demonstrated significantly improved accuracy and good predictive capabilities.

## 1. Introduction

Open-pit mining has advantages such as lower costs and higher recovery rates. While it can generate substantial economic benefits, it also leads to vegetation degradation in mining areas and pollution of the surrounding environment. Continued mining activities may cause ground subsidence, collapses, landslides, and other hazards, posing threats to production and the safety of people and property in mining regions [1,2,3,4,5,6,7,8,9]. Therefore, extensive ground subsidence monitoring in mining areas is crucial for ensuring normal production activities and safeguarding the safety of people and property [10].

Currently, surface deformation monitoring typically utilizes traditional instruments for elevation and distance measurements, such as conventional precise leveling and RTK surveying methods [11,12,13]. However, these methods require significant manpower and resources, and the monitoring data obtained are point-specific, unable to accurately reflect the overall condition of slopes. The development of Interferometric Synthetic Aperture Radar (InSAR) technology has progressed rapidly. Compared to traditional methods, InSAR offers advantages such as high efficiency and high accuracy. It has been widely applied in regional surface deformation monitoring, landslide prediction, earthquake deformation, and other fields [14]. Time-series InSAR technology effectively overcomes issues such as temporal and spatial decorrelation, atmospheric delays, and terrain effects on differencing, enabling high-precision surface deformation measurements. It facilitates continuous deformation monitoring over time and can simultaneously monitor large areas and multiple targets, offering advantages like low cost, high sampling density, and all-weather monitoring capability. Effective time-series deformation monitoring has gradually become the primary method for large-scale surface deformation monitoring [15]. Liu et al. [16] conducted an investigation using Permanent Scatterer Interferometry (PSI) and Small Baseline Subset (SBAS) techniques to analyze mining-induced subsidence in Sanshan Island, China. Their InSAR results showed that both methods identified similar subsidence patterns and areas, with a maximum Line-of-Sight (LOS) subsidence rate of approximately 49 mm/year. They found that PSI generally provided higher subsidence values compared to SBAS results when analyzing subsidence displacements at representative locations within the mining area. In another study by Gourmelen et al. [17] in a mining area in the Crescent Valley, NV, USA, SBAS-InSAR was employed to measure deformation velocities. Their findings indicated that the observed LOS displacement of up to 25 cm was caused by both vertical and horizontal deformations. They also detected a horizontal displacement rate of approximately 8 mm/year at a distance of 10 km from GPS stations. These studies demonstrate the application of advanced InSAR techniques in monitoring mining-induced subsidence and deformation velocities, highlighting their capability to provide detailed and precise measurements over large areas and varying terrain conditions.

The InSAR technique based on single-orbit SAR data can only capture one-dimensional deformation information along the LOS. When the deformation direction is parallel to the LOS, monitoring is most effective. However, if the deformation direction is perpendicular to the LOS, the InSAR technique is unable to detect any deformation. Surface deformation occurs in three-dimensional space, and one-dimensional deformation cannot accurately reflect the true state of the deforming body. Therefore, conducting two-dimensional and three-dimensional deformation studies based on InSAR technology is necessary and practically significant. Gray et al. [18] used D-InSAR technology to obtain one-dimensional deformation fields in the LOS direction from both ascending and descending orbits in the northern part of Ellesmere Island, Canada. By establishing a deformation model based on the spatial characteristics observed by SAR satellites, they combined the two one-dimensional deformation fields to derive high-precision two-dimensional deformation fields in the vertical and east–west directions. Samsonov et al. [19] utilized Multidimensional Small Baseline Subset InSAR (MSBAS-InSAR) technology to process SAR data with different parameters, extracting the surface two-dimensional deformation rate field in the Virunga volcanic region, Congo. Shahzad et al. [20] used InSAR Point Target Analysis (IPTA) technology to compute the surface two-dimensional deformation field in Abbottabad, Pakistan, using ascending and descending orbits Sentinel-1A data. These studies demonstrate that combining Sentinel-1A ascending and descending orbit data is sufficient to compute the surface two-dimensional deformation field, and this approach has been widely adopted in deformation analysis studies.

To mitigate potential deformation threats in mining areas, it is crucial to conduct predictive research on surface deformation. In recent years, machine learning has become a common method for predicting subsidence in mining areas. Common models include Support Vector Machines (SVM), BPNN, Convolutional Neural Networks (CNN), and Recurrent Neural Networks (RNN). These models can be broadly categorized into two types: single-model predictions and hybrid approaches. Ma et al. [21] proposed a method for predicting mine subsidence using InSAR technology combined with Long Short-Term Memory (LSTM) networks. The prediction results showed a maximum absolute error of less than 2 cm and a maximum relative error of less than 6%, indicating that integrating InSAR technology with the LSTM algorithm is an effective and robust method for predicting mine subsidence. Chen et al. [22] used LSTM to establish a prediction model based on time-series InSAR deformation data. They compared the results with RNN. The comparison indicated that LSTM provided more accurate predictions at the point scale than RNN.

The other category involves using composite models or optimized models for predictive research. Ding et al. [23] developed a settlement monitoring and dynamic prediction model by combining InSAR technology with the Golden Section Method-Holt-Winters (GSM-HW) model. The experimental results demonstrated that the GSM-HW prediction model addresses the parameter optimization deficiencies of the single HW model, achieving maximum fitting and prediction accuracies of 96.9% and 98.4%, respectively. He et al. [24] proposed a unified Convolutional Neural Network with Peephole Long Short-Term Memory (CNN-PhLSTM) to predict surface deformation in the Jinchuan mining area in Gansu Province, China. The proposed model was evaluated using metrics such as MAE. This indicates that composite or optimized models generally offer higher accuracy than single models in subsidence prediction studies and have become widely used methods for surface subsidence prediction.

SBAS-InSAR, as a mature technology, has been widely applied in monitoring surface deformation in mining areas. Open-pit mining sites typically feature extensive mining slopes and waste dump slopes, where deformations occur in three-dimensional space. The LOS deformation results may not accurately reflect the true deformation state. Additionally, because surface deformation in open-pit mines often exhibits complex and nonlinear characteristics, neural networks, with their strong nonlinear mapping capabilities, are well-suited for handling highly complex information, unclear factor relationships, and ambiguous background knowledge. Models like CNN and RNN require large amounts of training data and substantial training time and computational resources, making them more complex to implement. In contrast, this study’s point data samples are fewer and structurally simpler, making the use of BPNN more straightforward and efficient. Given that the BPNN model tends to converge slowly and can easily get trapped in local minima [25], while GA and ABC algorithm optimizations have shown better performance [26,27], GA and ABC algorithms are selected to optimize the weights and thresholds of the BPNN model to improve prediction accuracy.

In summary, this paper utilizes SBAS-InSAR technology to process Sentinel-1A ascending and descending orbit images covering the study area. Based on the cumulative subsidence values obtained from these orbits, the paper conducts a two-dimensional decomposition study of surface deformation in mining areas. Subsequently, the study predicts subsidence at characteristic points using BPNN models, namely the GA-BP and ABC-BP models, comparing their predictions with those of a standalone BPNN model to analyze the reliability and feasibility of the two optimization algorithms used in this research. Furthermore, the study summarizes the impact of rainfall on surface subsidence, discusses the differences between prediction methods based on various influencing factors, and provides insights that could aid in the identification and prevention of subsidence hazards in open-pit mining areas.

## 2. Materials and Methods

### 2.1. Study Area

Anning City is located in the northwest of Kunming, Yunnan Province, China, just 32 km from the main urban area of Kunming. It borders the Xishan District of Kunming to the northeast, the Jinning District of Kunming to the southeast, and the Yimen County of Yuxi City and Lufeng City of Chuxiong Prefecture to the west. Covering an area of approximately 1301.8 km^2^, Anning is a crucial passage to cities such as Chuxiong, Dali, Baoshan, Lijiang, and Shangri-La. The overall terrain within the city is relatively flat, with most areas having slopes of less than 20°. The highest elevation is 2617.7 m, and the lowest is 1690.2 m, with the general topography sloping from south to north, resulting in a significant relative elevation difference.

**Figure 1:**
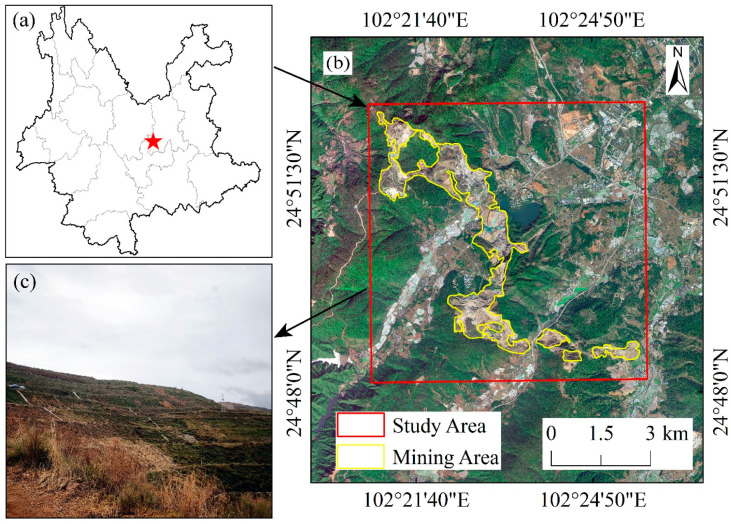
Overview map of the study area. (**a**) The approximate location of the study area (The red star) in Yunnan Province, China; (**b**) The extent and specific positioning of the mining area; (**c**) A field photo of a slope at an abandoned soil disposal site in the mining area.

Anning City is rich in mineral resources, with phosphate, iron, salt, and geothermal water being the primary resources. The mining economy, based on these resources, forms the foundational and pillar industries for Anning’s industrial and tourism sectors. The concentrated phosphate mining area is located southwest of Anning City, covering approximately 200.43 hectares, with coordinates ranging from 102°20′57″ to 102°25′41″ E and 24°48′29″ to 24°52′41″ N. The specific location is shown in Figure 1.

In terms of topographical features, the mining area is situated in the central Yunnan Plateau, within the Dianchi Rift Basin, characterized by dissected middle mountain landforms predominantly shaped by structural erosion. Climatically, the area experiences a subtropical highland monsoon climate due to its low latitude and high altitude, marked by distinct wet and dry seasons, concentrated rainfall, simultaneous occurrence of rain and heat during the same season, and small annual temperature variations but large diurnal temperature differences.

**Figure 2:**
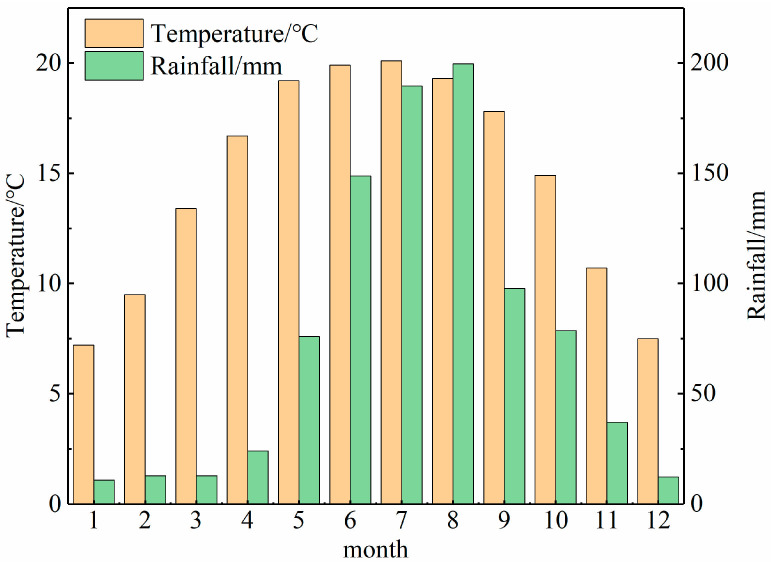
Average monthly temperature and precipitation over the years.

According to data from the Anning Meteorological Bureau, the average annual temperature in the mining area is 14.7 °C, with ample and evenly distributed sunshine. The hottest month is July, with an average temperature of 20.1 °C, while the coldest month is January, with an average temperature of 7.2 °C. The average ground temperature over the years is 18 °C, with an average of 225 frost-free days per year. The average annual rainfall is 886.5 mm, ranging from a maximum of 1161.8 mm to a minimum of 553.9 mm. The rainy season (May to October) accounts for 87% of the annual precipitation, while the dry season (November to April) contributes 13%, as shown in Figure 2.

The area exhibits intense structural activity, resulting in fragmented rock formations due to both structural movements and weathering effects. Despite relatively good vegetation cover, surface water easily infiltrates downwards, triggering geological hazards such as land subsidence and collapse landslides. Human engineering activities within the region, notably mining operations and road construction, contribute significantly to slope instability along weak structural planes, posing challenges for engineering projects.

### 2.2. Datasets

#### 2.2.1. Sentinel-1A Synthetic Aperture Radar Data

The Sentinel-1 satellite carries a C-band synthetic aperture radar and is part of the European Space Agency’s Copernicus program (GMES) [28]. Comprising two satellites, Sentinel-1A and Sentinel-1B, it provides all-weather, day-and-night Earth observation capabilities, continuously capturing global imagery with a revisit cycle of 12 days.

This study utilizes a total of 91 scenes of Sentinel-1A Single Look Complex (SLC) data, including 46 ascending orbit scenes, spanning from 20 May 2020, to 17 May 2023, and 45 descending orbit scenes, spanning from 10 May 2020, to 31 May 2023. The imaging mode is IW, with VV polarization, a range resolution of 5 m, and an azimuth resolution of 20 m. The specific dates of the ascending and descending orbit images are listed in Table 1 and Table 2.

#### 2.2.2. Digital Elevation Model (DEM)

The Shuttle Radar Topography Mission (SRTM), a joint effort by NASA and the National Geospatial-Intelligence Agency (NGA), was conducted aboard a spacecraft launched on 11 February 2000. Over 222 h and 23 min, it covered latitudes from 60° north to 60° south, producing radar image data that covers over 80% of the Earth’s land surface, totaling more than 11.9 million square kilometers. The data volume collected by the SRTM system amounted to approximately 9.8 terabytes. After over two years of processing, a DEM was created. Starting from 2003, these data products have been publicly available, continually revised and improved. The latest version, processed by CIAT (International Center for Tropical Agriculture) using advanced interpolation algorithms, effectively fills gaps in the original 90-m resolution dataset. SRTM terrain data is categorized into SRTM1 and SRTM3, representing 30-m and 90-m resolution data, respectively. For this experiment, NASA’s SRTM-DEM with 30-m resolution was utilized to mitigate terrain phase effects [29].

#### 2.2.3. POD Precise Orbit Ephemerides

Orbital information is crucial in InSAR data processing, playing a vital role in data preprocessing. Residual fringes in interferograms contain orbital errors, which can be effectively removed using precise satellite orbital data. POD Precise Orbit Ephemerides is the most accurate orbital data product, but it has a release delay of 21 days. A file is generated daily, with positioning accuracy better than 5 cm [30]. Given the large time span and high accuracy requirements, this study uses POD Precise Orbit Ephemerides corresponding to the imagery to improve orbital accuracy and remove orbital errors, yielding more precise monitoring results.

#### 2.2.4. Generic Atmospheric Correction Online Service (GACOS) Data

GACOS utilizes the Iterative Tropospheric Decomposition (ITD) model to separate vertically stratified components and horizontally correlated turbulent components from the total tropospheric delay. It generates high spatial resolution water vapor delay maps, removing atmospheric noise phases, which are used to correct InSAR measurements and other applications [31]. This study selected 91 GACOS data sets that completely cover the study area, corresponding to the dates of SAR images from ascending and descending orbits, aiming to mitigate the atmospheric effects on InSAR monitoring results.

#### 2.2.5. Rainfall Data

The rainfall data used in the Section 4 was downloaded from https://rp5.ru/ (accessed on 26 June 2024), where the original data consists of precipitation values measured at 3-h intervals. To align these values with the time series of settlement at prediction points, this study aggregated the precipitation values on a monthly basis. This yielded a dataset of monthly precipitation totals for the study area from May 2020 to May 2023, covering a span of 37 months.

### 2.3. Principles of Small Baseline Subset InSAR Technology

Berardino et al. [32] introduced the SBAS-InSAR technique in 2002, which effectively reduces phase noise and errors, mitigates spatial decorrelation, adds redundant observations, and enables joint solving to obtain temporal deformation sequences for deformation information. Assume that *S* images completely covering the study area are acquired over the time period from $t_{1}$ to $t_{s}$. One of these images is selected as the super master image. Then, based on parameters such as spatial baseline and temporal baseline, *N* differential interferograms are generated, where *N* satisfies the following formula:

(1) $$\frac{S}{2}\ll N\ll \frac{S(S-1)}{2}$$

For two images taken at times $t_{A}$ and $t_{B}(t_{A}$ < $t_{B})$, assume that the *i*-th differential interferogram (*i* = 1, 2, …, *N*) is generated. After removing or mitigating the error phase influences other than the deformation phase and assuming the deformation rates between different interferograms are $v_{k,k+1}$, the deformation phase for the period from $t_{A}$ to $t_{B}$ is given by Equation (2):

(2) $$\delta \varphi_{i}(x,r)=\frac{4\pi }{\lambda }\sum_{k=t_{A}}^{t_{B}-1}\left(t_{k+1}-t_{k}\right)v_{k,k+1}$$

In the equation, λ represents the wavelength; for a given pixel in the *i*-th differential interferogram with azimuth and range coordinates (x,r), $\delta \varphi_{i}(x,r)$ denotes the interferometric phase of that pixel.

**Figure 3:**
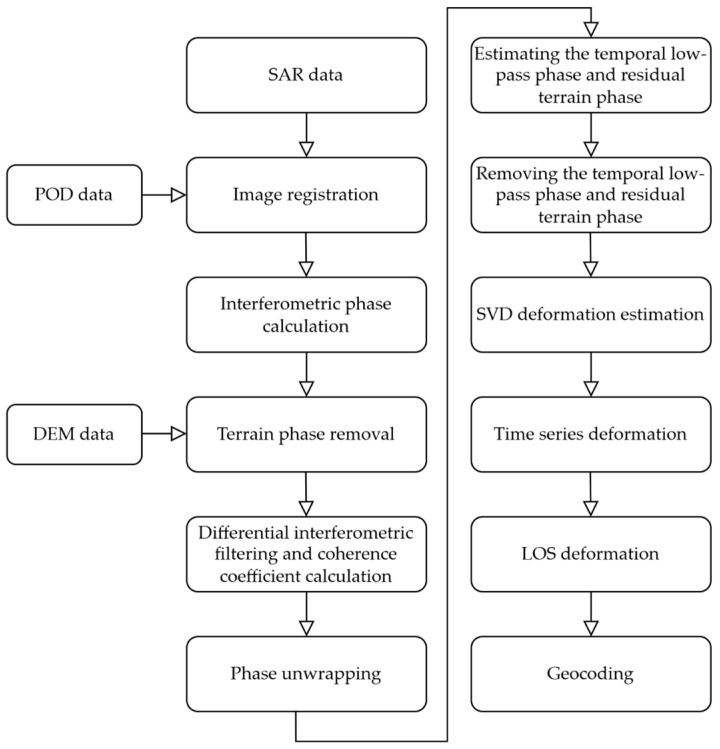
SBAS operational principle flowchart.

Utilizing Singular Value Decomposition (SVD) to analyze *N* differential interferograms, thereby deriving deformation velocity values for different SAR image time series. The specific processing steps are shown in Figure 3.

### 2.4. Principles of Two-Dimensional Deformation Decomposition

Conventional InSAR technology monitors surface deformation, with its observations representing the deformation occurring between the acquisition times of two images. This deformation value only represents the projection of deformation in various directions of the surface onto the radar line of sight and does not reflect the actual state of surface deformation. Due to the near-polar orbit characteristics of existing on-orbit radars, their ability to monitor north–south direction deformation is relatively weak. Therefore, by ignoring the north–south deformation component in the radar line of sight direction and combining ascending and descending InSAR observations, it is possible to derive the two-dimensional surface deformation field in the vertical and horizontal east–west directions [33].

**Figure 4:**
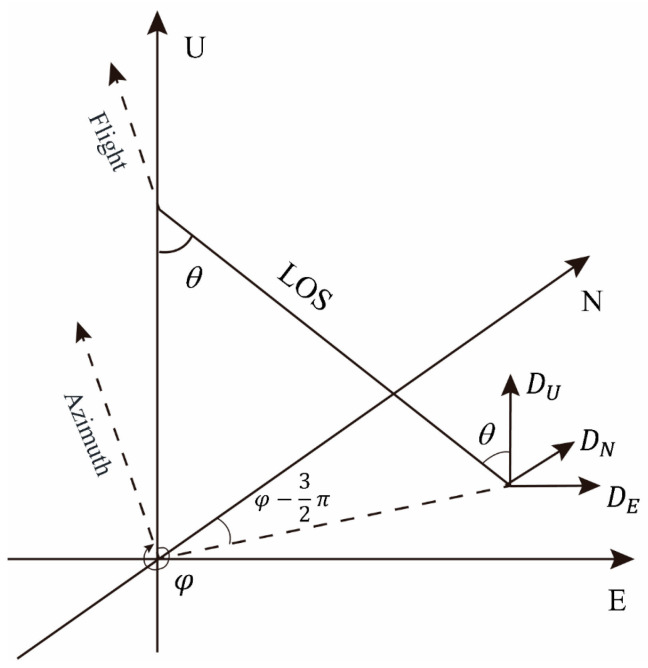
3D decomposition of LOS deformation.

To obtain the two-dimensional surface deformation field, the relationship between InSAR observations and the vertical and horizontal east–west deformation components is first analyzed. The spatial geometric characteristics of SAR satellite observations are shown in Figure 4.

From the above figure, it can be understood that the relationship between LOS deformation and three-dimensional deformation satisfies Equation (3) [34]:

(3) $$\begin{array}{l} D_{LOS}=D_{U}\cos\theta -D_{N}\sin\theta \cos(\varphi -\frac{3}{2}\pi )-D_{E}\sin\theta \sin(\varphi -\frac{3}{2}\pi )\\ =\left[\cos\theta -\sin\theta \cos(\varphi -\frac{3}{2}\pi )-\sin\theta \sin(\varphi -\frac{3}{2}\pi )\right]\left[\begin{array}{c} D_{U}\\ D_{N}\\ D_{E} \end{array}\right] \end{array}$$

In this equation, $D_{LOS}$ represents the Line-of-Sight deformation, $D_{U}D_{N}D_{E}$ denote the vertical, north–south, and east–west deformations, respectively. θ is the radar incidence angle, φ is the satellite heading angle, and $\varphi -\frac{3}{2}\pi$ is the projection direction of the LOS in the horizontal plane.

Currently, radar satellites in orbit tend to fly with their flight direction close to due north. Due to satellite system configurations, the imaging geometry diversity of radar satellite data is limited, resulting in InSAR monitoring being insensitive to north–south deformations. To ensure the accuracy of least squares calculations, a common practice is to neglect the north–south deformation in the above model and solve for vertical and east–west deformations using data from two orbits satellites.

Equation (3) represents the decomposition of LOS deformation in three-dimensional directions for a single orbit. When joint ascending and descending orbits observation data are used for two-dimensional surface deformation decomposition, it can be formulated in matrix form as shown in Equation (4) [34]:

(4) $$\left[\begin{array}{c} D_{LOS1}\\ D_{LOS2} \end{array}\right]=\left[\begin{array}{cc} \cos\theta_{1} & -\sin\theta_{1}\sin(\varphi_{1}-\frac{3}{2}\pi )\\ \cos\theta_{2} & -\sin\theta_{2}\sin(\varphi_{2}-\frac{3}{2}\pi ) \end{array}\right]\left[\begin{array}{c} D_{U}\\ D_{E} \end{array}\right]$$

### 2.5. Principle of Prediction Models

#### 2.5.1. Back Propagation Neural Network

The BPNN is an algorithm that resolves weight adjustments in multi-layer neural networks through error backpropagation, making it one of the most widely applied neural network models. Its network structure comprises input layers, hidden layers, and output layers, with the learning process consisting of two main parts: forward propagation of information and backward propagation of errors [35]. The specific parameters of the algorithms are shown in Table 3.

**Figure 5:**
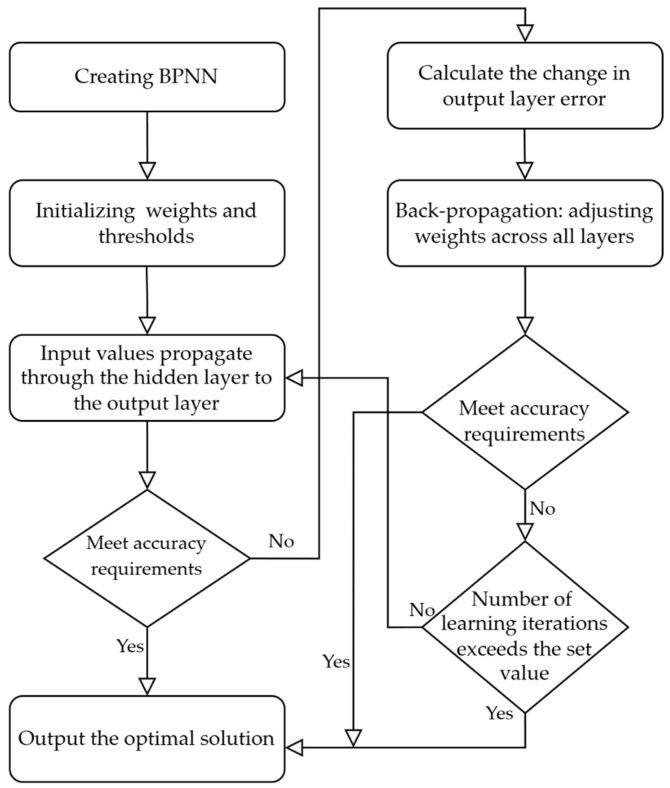
Processing workflow of the BPNN model.

During forward propagation, input information passes sequentially from the input layer through the hidden layers to the output layer. If the output layer does not yield the desired result, the error variation of the output layer is computed, and gradient descent is employed to initiate backward propagation. Through the neural network, errors are propagated back along the original path, thereby further modifying the weights of neurons at each layer. Once this process completes, input information is reintroduced into the network via the input layer, and the above process is repeated. Iterative adjustments to neuron weights continue until the sum of squared errors of the neural network is minimized to meet the error target or reaches a preset iteration limit (Figure 5).

#### 2.5.2. BPNN Optimized by Genetic Algorithm

**Figure 6:**
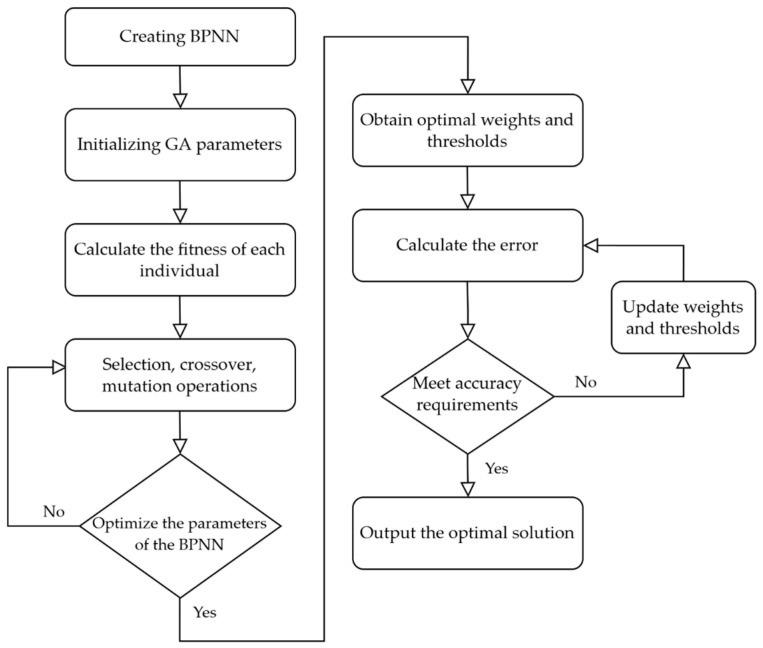
Explain the optimization steps in GA-BP.

The Genetic Algorithm is a population-based search optimization algorithm [36]. It uses binary encoding to represent numerous optimization variables and combines the binary-encoded strings of these variables to form chromosomes. According to the principle of “survival of the fittest”, chromosomes are randomly selected from the initial population to undergo crossover and mutation operations. Through successive iterations of evolution, the newly generated population eventually contains the optimal solution or an individual close to the optimal solution. The optimization process is illustrated in Figure 6. The specific parameters of the algorithms are shown in Table 4.

The specific steps are as follows:

(1) Construct the BPNN model, determine the number of nodes in each layer, and normalize the input data.
(2) Set the main parameters of the GA optimization algorithm, including the maximum number of generations (G), crossover probability ($P_{c}$), mutation probability ($P_{m}$), and population size ($N_{P}$).
(3) Calculate the fitness of each individual and use selection, crossover, and mutation principles to optimize the initial weights and thresholds of the BPNN.
(4) Ensure that the initial weights and threshold indicators of the BPNN model are optimal, then perform gradient descent to search the solution space for these parameters and update the weights and thresholds.
(5) If the accuracy condition is met, output the optimal solution; otherwise, proceed to the next iteration until the optimal solution is found.

#### 2.5.3. BPNN Optimized by Artificial Bee Colony Algorithm

**Figure 7:**
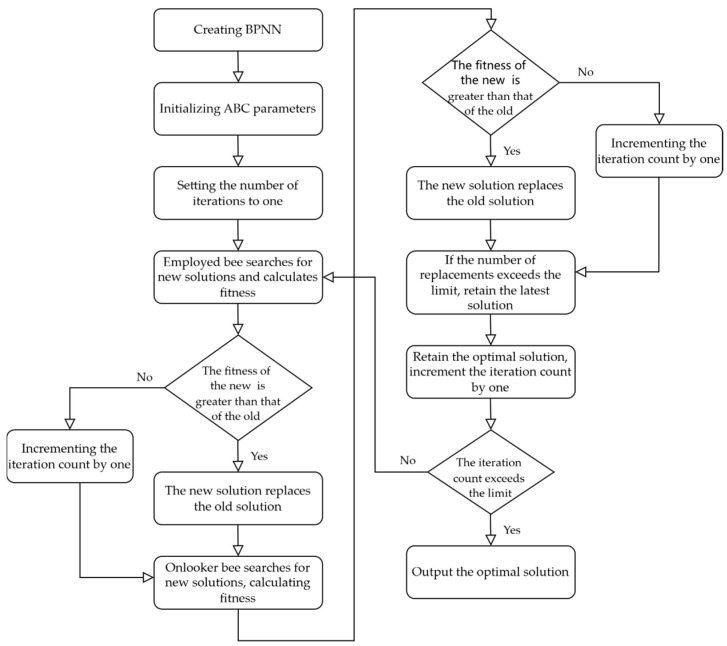
Explain the optimization steps in ABC-BP.

The Artificial Bee Colony algorithm is a global optimization search algorithm inspired by the efficient foraging behavior of bees in finding high-quality nectar sources. The optimization process is divided into three phases: the employed bee phase, the onlooker bee phase, and the scout bee phase [37]. Bees and honey sources are the two core elements of the ABC model. Scout bees are tasked with finding honey sources, while employed bees and onlooker bees are responsible for exploiting these sources. Employed bees locate honey sources, assess their size and quantity, and release signals marking paths to attract more bees for exploitation. Onlooker bees mark and search for new honey sources nearby, focusing on higher-quality ones. If a honey source is exploited consistently for a period, new scout bees are dispatched to find alternative sources, maximizing honey collection efficiency. In optimization problems, honey source locations represent potential solutions, and the quantity of honey sources corresponds to their fitness as solutions. Bees search near the hive for honey sources; rich sources signal positively to guide more bees, while poor sources signal negatively to deter them. The abundance of artificial honey sources indicates better solution quality, guiding the bee swarm toward optimal solutions. The optimization process is illustrated in Figure 7. The specific parameters of the algorithms are shown in Table 5.

The specific steps are as follows:

(1) Construct the BPNN model, determine the number of nodes in each layer, and normalize the input data.
(2) Set the parameters of the ABC algorithm, including population size (*N*), maximum number of iterations (*M*), upper bound (*x_j_*) and lower bound (*y_j_*) of the search space, and the dimension of the solution (*D*).
(3) Set the initial weights and thresholds of the BPNN according to the solution dimension (*D*). Define the position of the *i*-th employed bee’s random search solution as $d_{ij}$, and search for new solutions in its vicinity and record the positions.
  (5) $$d_{ij}=n_{j}+\mathrm{rand}(0,1)(x_{j}-n_{j})$$
  In the equation, $n_{j}$ represents the lower bound of the search range for the *j*-th element of each solution while $x_{j}$ represents the upper bound of the search range for the *j*-th element of each solution.
(4) Calculate the fitness value ($f_{i}$) of the solution and use the roulette wheel algorithm to compute the probability ($p_{i}$) of each solution being selected, choosing the optimal solution.
  (6) $$p_{i}=\frac{f_{i}}{\sum_{i=1}^{N}f}$$
(5) Onlooker bees use a greedy algorithm to update the position $e_{ij}$ of the previously best solution. If no better solution is found in the vicinity, increment the record count for that solution.
  (7) $$e_{ij}=d_{ij}+\varphi (d_{ij}-d_{kj})\left(k=1,2\cdots ,N;k\neq i\right)$$
  In the equation, φ is a random number within the range of [−1, 1], and $d_{kj}$ represents a neighboring solution.
(6) Save the optimal solution and determine whether to discard a solution based on the number of times it has been recorded. If a solution is discarded, the scout bees randomly generate a new solution using Equation (5).
(7) If the number of times a solution is recorded is greater than or equal to the maximum number of iterations, output the optimal solution. Otherwise, proceed to the next iteration until the optimal solution is found.

### 2.6. Evaluation Metrics

To verify the accuracy of surface subsidence predictions in mining areas and assess the reliability of the model, two evaluation metrics, MAE and RMSE, will be employed in the experiments.

(1) MAE:

(8) $$MAE=\frac{1}{m}\sum_{i=1}^{m}\left|y_{i}-y_{i}^{*}\right|$$

(2) RMSE:

(9) $$RMSE=\sqrt{\frac{1}{m}\sum_{i=1}^{m}{\left(y_{i}-y_{i}^{*}\right)}^{2}}$$

In Equations (8) and (9), where m is the number of samples, $y_{i}$ represents the actual values of the samples, and $y_{i}^{*}$ denotes the predicted values of the samples. MAE is used to evaluate the accuracy of the model, preventing the problem of error cancellation among discrepancies. A smaller MAE indicates a better fit between predicted and actual values, thus indicating higher model accuracy. On the other hand, RMSE assesses model accuracy by measuring the magnitude of deviations between predicted and actual values. A smaller RMSE suggests less deviation between predicted and actual values.

## 3. Results

### 3.1. Data Processing

To systematically study the characteristics of surface deformation in the southwestern phosphate mining area of Anning City, this paper utilizes SARscape 5.6.2 software to conduct SBAS-InSAR processing. A total of 91 scenes of Sentinel-1A data were selected, with 46 ascending orbit scenes spanning from 20 May 2020, to 17 May 2023, and 45 descending orbit scenes spanning from 10 May 2020, to 31 May 2023. By setting the temporal and spatial baselines, 178 interferometric pairs were generated from the ascending orbit data, and 174 pairs from the descending orbit data. GACOS data were imported, and interferograms were generated using the Delaunay MCF unwrapping method and Goldstein filtering. If the coherence of the differential interferograms obtained after SBAS-InSAR processing is high, the sequence information is geocoded after orbit refinement, re-flattening, first inversion, and second inversion steps, resulting in deformation rate values in the LOS direction for both ascending and descending orbits.

### 3.2. Distribution Characteristics of Subsidence in Mining Areas

**Figure 8:**
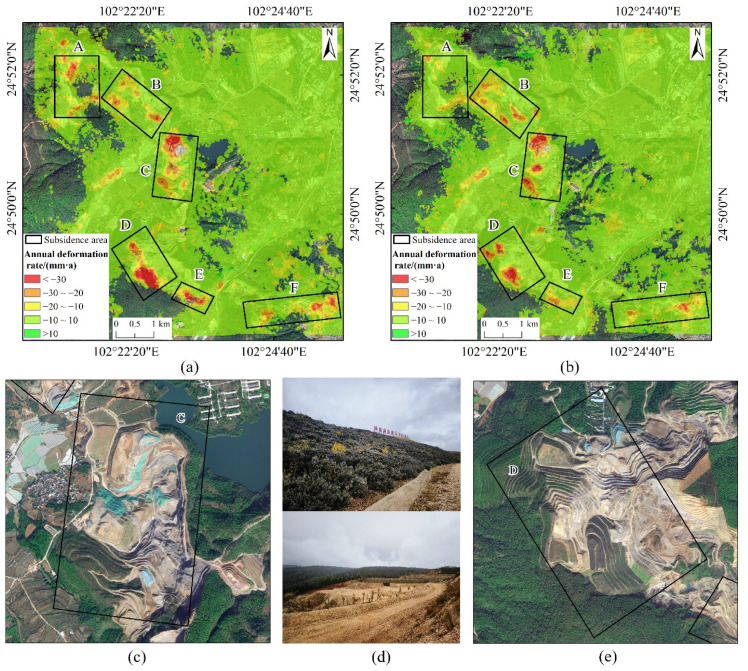
Average annual rate of deformation. (**a**) Ascending orbit deformation rate; (**b**) Descending orbit deformation rate; (**c**) Optical image of subsidence Area C; (**d**) Slopes of abandoned soil fields within subsidence Areas A and B; (**e**) Optical image of subsidence Area D.

Within the mining area, there are approximately six major subsidence zones, designated as A, B, C, D, E, and F, as shown in Figure 8.

Based on the results from the ascending orbit data, subsidence in zone D exhibits the most severe conditions, with a maximum subsidence rate of approximately 68 mm/year. Other areas show lower maximum subsidence values compared to zone D: zone A reaches a maximum subsidence rate of 42 mm/year, zone B reaches 39 mm/year, zone C reaches 51 mm/year, zone E reaches 46 mm/year, and zone F reaches 47 mm/year, as depicted in Figure 3. From the descending orbit data, zone C shows the most severe subsidence conditions, with a maximum subsidence rate of approximately 53 mm/year. Other areas exhibit lower maximum subsidence values compared to zone C: zone A reaches a maximum subsidence rate of 30 mm/year, zone B reaches 38 mm/year, zone D reaches 52 mm/year, zone E reaches 39 mm/year, and zone F reaches 44 mm/year. Overall, zones C and D are identified as the most severely affected subsidence areas within the entire study area, with subsidence funnels located primarily in the overburden disposal areas and reclamation zones. Optical imagery reveals that significant restoration efforts have been undertaken in these areas, with most showing varying degrees of vegetation cover except for a few areas where the ground remains exposed.

**Figure 9:**
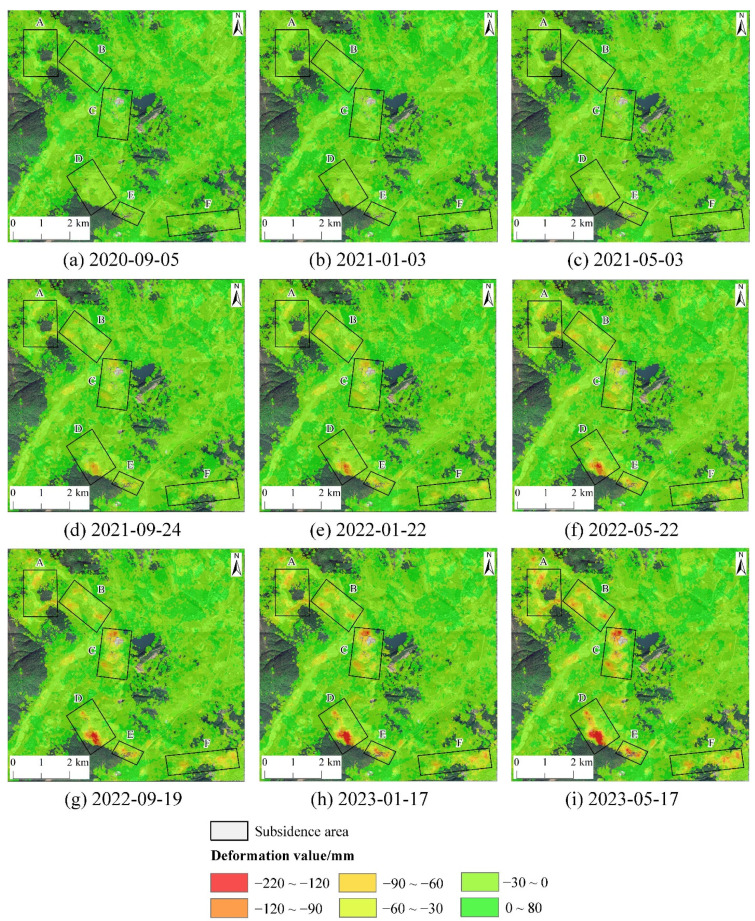
The accumulated deformation value from ascending orbit.

The temporal cumulative deformation from the ascending orbit images is shown in Figure 9. From Figure 9, it is evident that each subsidence zone began to exhibit noticeable subsidence and gradually expanded from 20 May 2020, to 22 May 2022. The cumulative subsidence within these zones showed an increasing trend during this period. From 22 May 2022, to 17 May 2023, the expansion of the subsidence zones gradually halted, but the cumulative subsidence continued to increase. Among these, zone D recorded the highest maximum subsidence value, reaching up to 211 mm. Zone A had a maximum subsidence value of 130 mm; zone B, 121 mm; zone C, 147 mm; zone E, 133 mm; and zone F, 157 mm.

**Figure 10:**
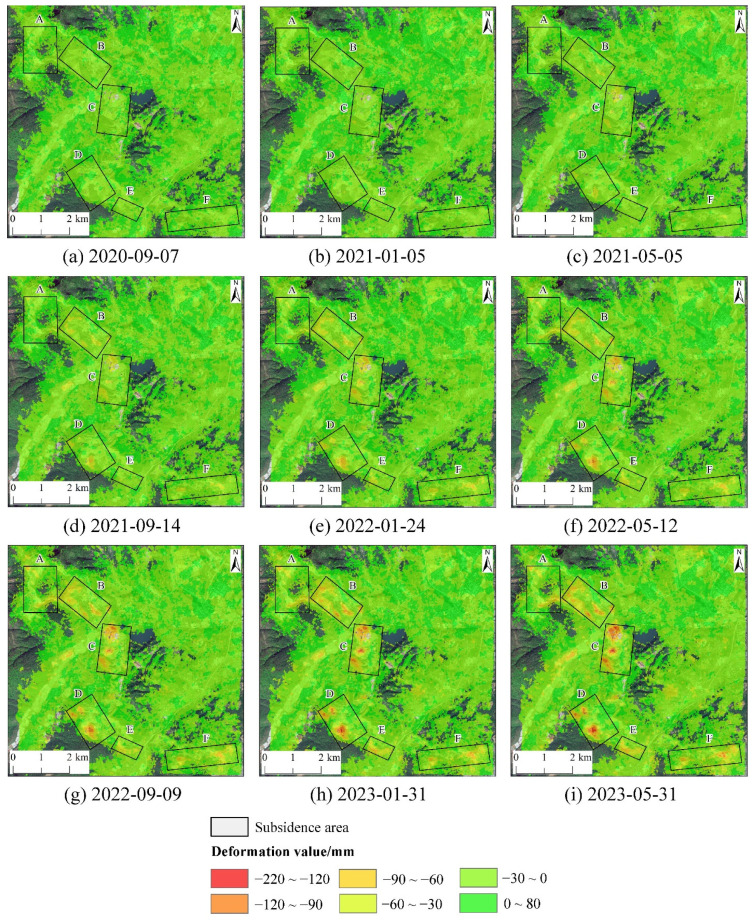
The accumulated deformation value from descending orbit.

The temporal cumulative deformation from the descending orbit images is shown in Figure 10. From Figure 10, it is clear that each subsidence zone began to exhibit noticeable subsidence and gradually expanded from 10 May 2020, to 12 May 2022. The cumulative subsidence within these zones showed an increasing trend during this period. From 12 May 2022, to 31 May 2023, the expansion of the subsidence zones gradually ceased, but the cumulative subsidence continued to increase. Among these, zone C recorded the highest maximum subsidence value, approximately 158 mm. Zone A had a maximum subsidence value of around 110 mm; zone B, around 115 mm; zone D, around 141 mm; zone E, around 105 mm; and zone F, around 124 mm.

### 3.3. Two-Dimensional Decomposition of Surface Deformation

**Figure 11:**
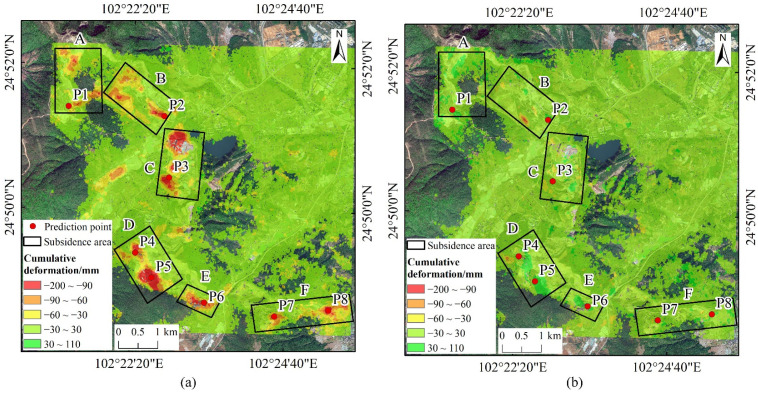
Results of 2D deformation decomposition (**a**) Cumulative vertical deformation; (**b**) Cumulative east–west deformation.

Surface 2D deformation inversion experiments were conducted using the sequential, cumulative deformation values obtained from ascending and descending orbit perspectives. MATLAB R2022a software was utilized, employing a cubic spline function method to interpolate descending orbit data based on time intervals from ascending orbit data. Using Equation (4), the sequential, cumulative deformation values in the vertical and east–west directions for the study area were computed. The decomposed results of the final cumulative deformation values in these two directions are presented in Figure 11.

Based on the vertical deformation results of the mining area, the region with the highest vertical deformation values remains in Area D, consistent with the results from the ascending orbit perspective, indicating it as the most severely subsided area. Specifically, Area A shows maximum vertical subsidence of 107 mm, Area B of 117 mm, Area C of 151 mm, Area E of 119 mm, and Area F of 121 mm, whereas Area D exhibits the highest vertical subsidence reaching 190 mm. Regarding the east–west deformation results of the mining area, the maximum eastward deformation observed is 106 mm, while the maximum westward deformation is 107 mm. Overall, Area D exhibits the most severe east–west deformation compared to other subsided areas, with a maximum westward deformation of 74 mm and a maximum eastward deformation of 102 mm. Specifically, in Area A, the maximum westward deformation is 62 mm and eastward deformation is 73 mm; in Area B, westward deformation is 54 mm and eastward deformation is 50 mm; in Area C, westward deformation is 60 mm and eastward deformation is 69 mm; in Area E, westward deformation is 49 mm and eastward deformation is 105 mm; in Area F, westward deformation is 56 mm and eastward deformation is 66 mm.

Using MATLAB R2022a, time series points were extracted from the vertical deformation raster data. One time series point was selected in each of the six major subsidence areas, labeled P1, P2, P3, P4, P5, P6, P7, and P8, as shown in Figure 11a.

**Figure 12:**
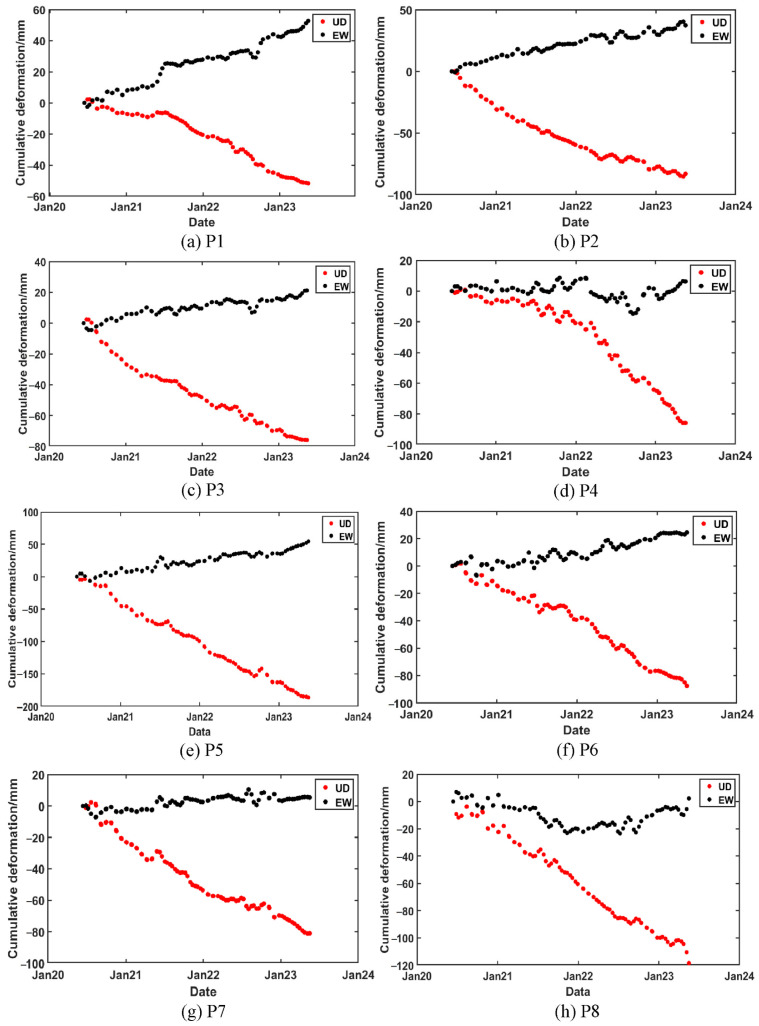
Time-series 2D deformation values of predictive points.

Figure 12 shows the 2D deformation values over time for the selected points. The vertical and east–west deformation trends for each point exhibit significant differences. Due to the varying degrees of subsidence, each point effectively reflects the subsidence trend in its respective area, providing good representativeness. Therefore, P1 through P8 can be used as predictive points for future subsidence analysis.

### 3.4. Analysis of Subsidence Prediction Results

**Figure 13:**
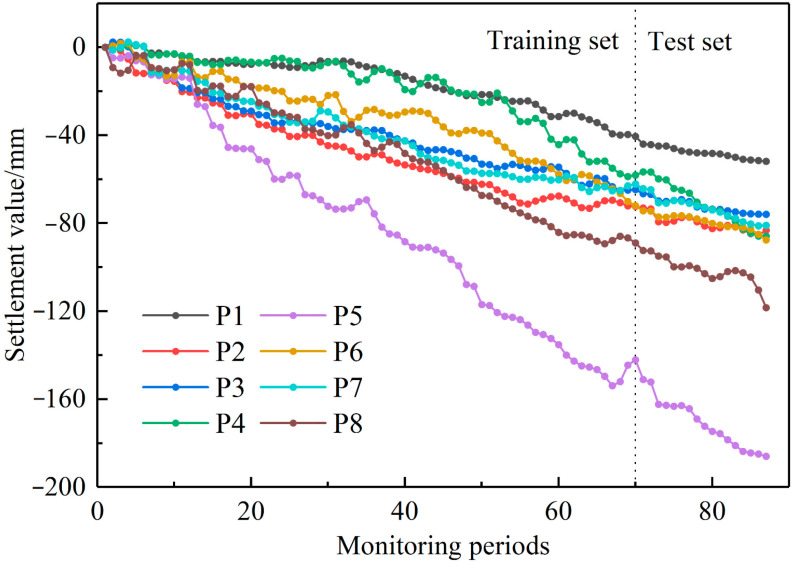
Time Series Settlement Results of Predictive Points.

Based on the vertical subsidence values from the time series in Figure 11 (corresponding to the red points), the 87-period data of the eight predictive points were split into training and testing sets in approximately an 8:2 ratio. The first 70 periods were used as the training set input for the prediction model, while the subsequent 17 periods formed the testing set, as illustrated in Figure 13.

To better reflect the error comparison between the monitored and predicted values for points P1 to P8, two metrics, MAE and RMSE, were employed to evaluate the accuracy and reliability of the model predictions, as shown in Table 6.

**Figure 14:**
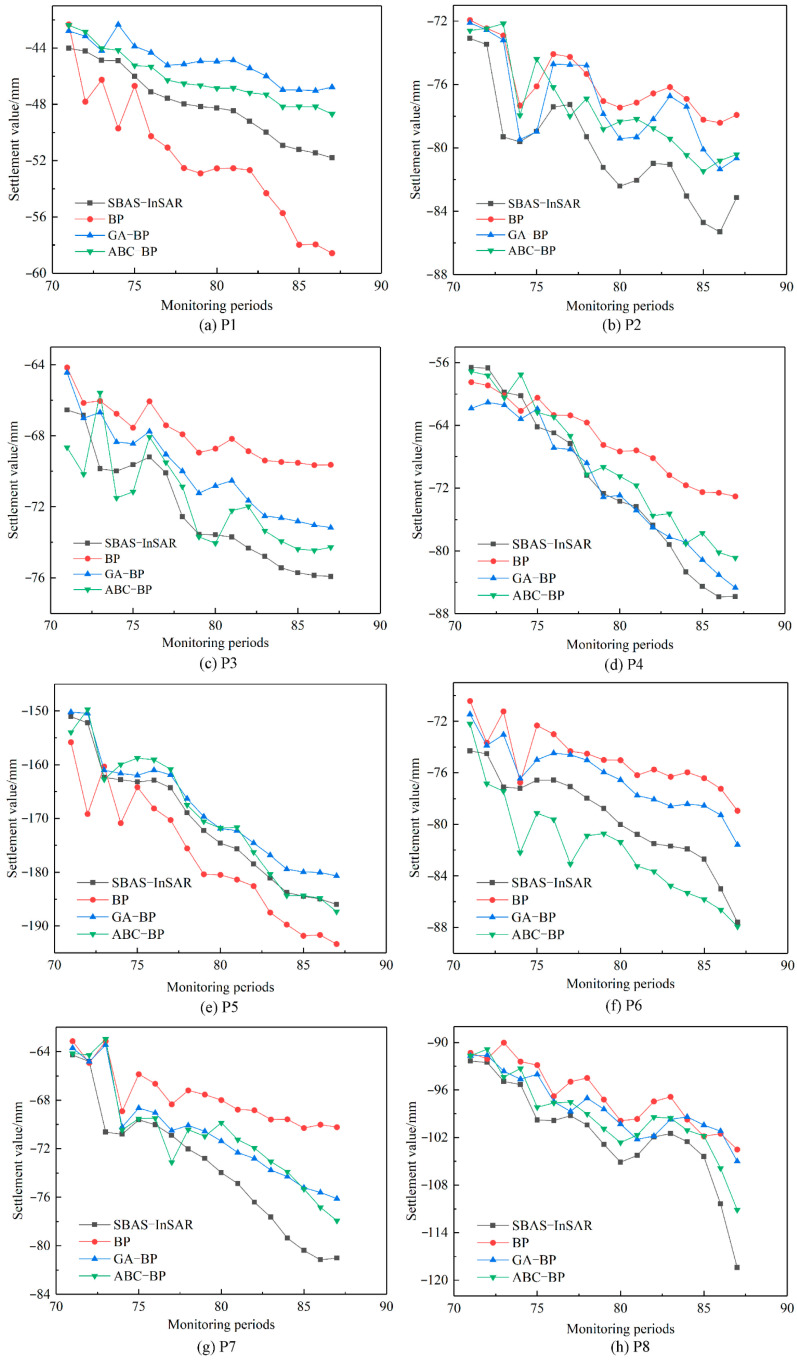
Comparison of prediction results of different models.

This study uses MATLAB R2022a software to conduct subsidence prediction experiments in mining areas. To compare and verify the predictive capability of the BPNN model, two optimized models, GA-BP and ABC-BP, were further used to predict eight characteristic points. The comparison between SBAS-InSAR monitoring values and the predicted values from the BPNN, GA-BP, and ABC-BP models is shown in Figure 14. Among the predictions for the eight characteristic points using the BPNN model, the predictions for points P1 and P2 are more accurate, with MAEs less than 4.3 mm and RMSEs less than 4.6 mm. However, the prediction accuracy for points P4 and P5 is relatively poor compared to points P7 and P8, with MAEs greater than 6.3 mm and RMSEs greater than 7.1 mm.

The predictions of the GA-BP and ABC-BP models for the eight characteristic points exhibit strong consistency with SBAS-InSAR monitoring results. Specifically, the GA-BP model achieves a maximum MAE of 3.10 mm and a maximum RMSE of 3.45 mm, while the ABC-BP model maintains an MAE below 2.92 mm and a maximum RMSE not exceeding 3.64 mm. Across all eight points, the GA-BP model averages an MAE of 2.67 mm and an RMSE of 2.93 mm, whereas the ABC-BP model averages an MAE of 2.32 mm and an RMSE of 2.79 mm. These averaged precision metrics indicate that the ABC-BP model performs slightly better, demonstrating superior predictive results compared to the GA-BP model.

In the predictions for the eight characteristic points, the BPNN models optimized by GA and ABC consistently outperform the traditional BPNN model in terms of accuracy and trend. This further validates that the GA-BP and ABC-BP models are reliable methods for subsidence prediction in mining areas, offering significant practical value.

## 4. Discussion

### 4.1. The Impact of Rainfall on Subsidence

Rainfall has a significant impact on surface stability, especially in open-pit mining areas and spoil tips. Rain increases soil moisture content, reducing friction between soil particles and causing the soil to soften, which increases its fluidity and plasticity. When soil moisture reaches a certain level, soil stability decreases. Heavy rainfall can also raise the groundwater level. In open-pit mining areas, if the groundwater level rises close to or above the surface, it increases the water pressure on surface and subsurface structures, thereby reducing the soil’s bearing capacity. This makes the area prone to surface subsidence, landslides, and collapses [38].

**Figure 15:**
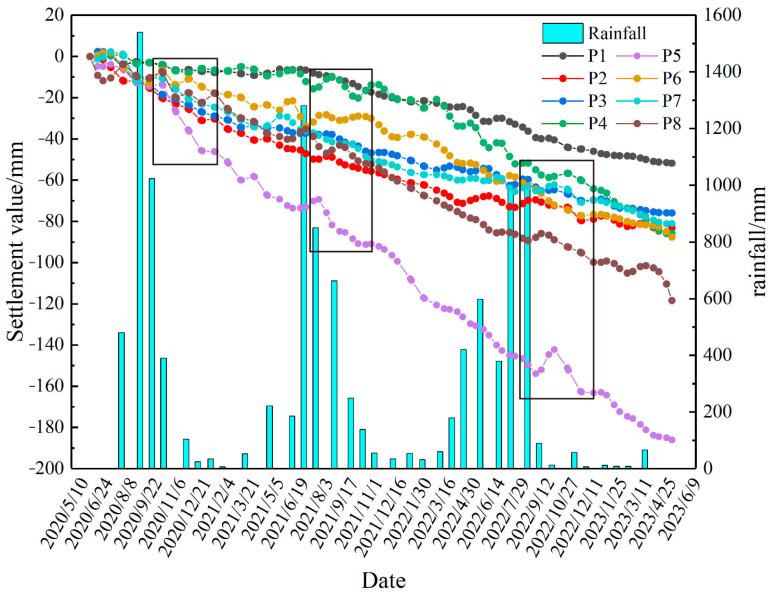
Subsidence Values at Prediction Points and Rainfall (The boxes in the figure indicate the lag effect of rainfall on surface deformation.).

To further elucidate the relationship between surface deformation and rainfall, this study conducts an overlay analysis of cumulative vertical deformation at eight feature points with rainfall data. As depicted in Figure 15, all selected subsidence points exhibit nonlinear sinking and show a strong correlation with rainfall. Deformation fluctuates in response to variations in rainfall, accelerating notably with increased precipitation. During periods of elevated rainfall, notably from June to August 2020, June to August 2021, and May to September 2022, surface deformation in the mining area intensifies significantly following peak rainfall events. This suggests that the impact of rainfall on surface deformation typically manifests after a certain delay, as water infiltrates the ground, altering soil moisture content and triggering expansion and contraction, thus resulting in surface deformation. Concurrently, rainfall replenishes groundwater, causing the water table to rise, and interactions between groundwater and the surface contribute to surface deformations such as uplift or subsidence. Therefore, the influence of rainfall on surface deformation often exhibits a lag effect [39].

Among the subsidence points, P3, P4, P5, P6, P7, and P8 show rapid subsidence. In contrast, the P1 and P2 subsidence points exhibit relatively gradual subsidence, but the subsidence rate sharply accelerates from May to September 2022. As seen in the figure, the study area experienced abundant rainfall from May to September 2022, with the highest monthly rainfall of 1009.9 mm occurring in August. This indicates that the excessive rainfall during this period caused the accelerated subsidence at the P1 and P2 points.

### 4.2. The Current Limitations of Study

#### 4.2.1. Potential Biases in Interferometric Synthetic Aperture Radar Monitoring Experiments

This study utilizes Sentinel-1A ascending and descending orbit images for surface subsidence monitoring in mining areas. However, Sentinel-1A data has low resolution. As SAR satellite data, such as LT-1 in the L-band and GF-3 in the C-band, becomes more abundant, applications for surface subsidence monitoring based on these SAR images need further exploration. Future research could benefit from higher-resolution SAR images to incorporate multi-source data. During interferometric processing, registration using SRTM DEM at 30-m resolution introduces DEM errors. The low resolution of the DEM limits data accuracy. Additionally, since SAR satellite image acquisition and DEM data retrieval occur at different times, there is a significant discrepancy between the referenced DEM elevation and actual elevation, which needs consideration in further studies.

In this study, the widely used SAR processing software SARscape was chosen. In future research, it would be beneficial to explore other mature software tools simultaneously to enhance monitoring accuracy through advancements in model algorithms and processing techniques. Moreover, within the mining area, there are numerous low-coherence regions where mature SBAS techniques still fail to detect many points. Subsequent research could address this issue using methods such as DS-InSAR.

The study did not collect concurrent leveling or GPS data for external accuracy validation. Acquiring external observational data corresponding to the study area would complement this aspect of the work. Additionally, the spatiotemporal analysis of subsidence areas in this study has certain limitations. Future efforts should involve gathering data on mining progress schedules to better support the analysis of monitoring results.

#### 4.2.2. Limitations of the Predictive Model and Directions for Future Research

Zhou et al. proposed a method for constructing prediction models from a multi-factor perspective [35]. They pointed out that existing prediction models overly rely on subsidence data. The principle of these models is to input the obtained subsidence data into a specific model to derive a prediction value, which does not achieve true prediction in the real sense. Therefore, in their research, they used SBAS-InSAR technology to process the study area, obtaining maps of subsidence regions and annual subsidence rates. They identified the influencing factors of surface subsidence in mining areas, performed a grey relational analysis between these influencing factors and the monitored values, and identified the optimal influencing factors. These factors were used as the input layer of the prediction network, with subsidence values as the output layer. This approach allowed them to construct a prediction function model for influencing factors and subsidence rates and to learn the optimal parameters through this model.

This predictive method considers the influence of multiple factors on subsidence but cannot forecast the multi-period subsidence trend at specific points within subsidence areas. This study attempted this predictive approach, but due to the small scope of the study area and the limited diversity in certain data (such as lithological data at 1:200,000 scale), there were only two distinct values within the mining area. This limitation resulted in overly homogeneous training samples, preventing precise prediction. Moreover, obtaining more accurate influencing factor data was hindered by various reasons. Consequently, the study opted to use subsidence values from time-series points as the training set for fitting and prediction. However, this approach overly relied on subsidence data from the same points, incapable of predicting other areas within the subsidence region and challenging for large-scale subsidence area forecasting. Therefore, finding a method to overcome the shortcomings of existing prediction models, integrating multiple influencing factors, and conducting long-term sequence forecasting remains a crucial challenge in current ground subsidence prediction efforts.

This study employed the traditional BPNN model, optimized using GA and ABC, resulting in more accurate predictions. Future research could consider using models such as LSTM for deeper time series prediction, exploring the predictive capabilities of different models to enhance prediction accuracy. As shown in Figure 10, with the increase in the number of monitoring periods, the differences between the predicted values of the three models and the InSAR monitoring values have significantly increased compared to earlier periods. When the number of prediction periods is too high, prediction accuracy declines. This indicates that the prediction model in this study is more suitable for short-term predictions, and models suitable for longer time series predictions require further exploration.

## 5. Conclusions

This study combined ascending and descending orbit data from Sentinel-1A to conduct surface deformation monitoring using SBAS-InSAR technology in the mining area. It obtained the annual average surface deformation rates and time series deformation results for the area. Based on the vertical cumulative deformation results derived from two-dimensional deformation decomposition, the study used BPNN and optimized models (GA-BP and ABC-BP) to predict subsidence values at deformation points. The following conclusions were drawn:

(1) The SBAS-InSAR monitoring results indicate that continuous subsidence has occurred in the study area since May 2020. By May 2023, six distinct deformation zones had been identified, all forming subsidence funnels. The ascending orbit results show that the D subsidence zone has the highest annual subsidence rate, approximately 68 mm/year, with a cumulative subsidence of about 211 mm. The descending orbit results indicate that the C subsidence zone has the highest annual subsidence rate, approximately 53 mm/year, with a maximum cumulative subsidence of around 155 mm. According to the vertical deformation results for the mining area, the D zone also exhibits the highest vertical deformation value, reaching 190 mm. The maximum vertical subsidence values in other areas range from 107 mm to 151 mm. The east–west deformation results for the mining area show that the D zone has the most severe east–west deformation compared to other subsidence zones, with the maximum westward deformation being 74 mm and the maximum eastward deformation being 102 mm. In other areas, the maximum westward deformation ranges from 49 mm to 62 mm, and the maximum eastward deformation ranges from 50 mm to 105 mm. Therefore, it is evident that both the vertical and east–west deformation values in the D subsidence zone are higher than those in other areas. Special attention should be paid to the stability of the surface in this zone during subsequent mining activities to prevent potential disasters.
(2) Based on the vertical cumulative subsidence values, neural network prediction models were used to forecast outcomes. It was found that the traditional BPNN model had maximum MAE and RMSE values of 6.41 mm and 7.58 mm, respectively. In contrast, the GA-BP model and ABC-BP model showed superior MAE and RMSE values. Specifically, the GA-BP model improved MAE values by 28% to 68% and RMSE values by 25% to 67% over the BPNN model. Similarly, the ABC-BP model increased MAE values by 32% to 63% and RMSE values by 28% to 60% over the BPNN model. Across all eight points evaluated, the GA-BP model averaged an MAE of 2.67 mm and an RMSE of 2.93 mm, while the ABC-BP model averaged an MAE of 2.32 mm and an RMSE of 2.79 mm, slightly outperforming the GA-BP model. These results indicate that the optimized BPNN prediction models are highly applicable for forecasting mining-induced subsidence, particularly when compared with decomposed vertical subsidence data, demonstrating their accuracy and suitability for use in mining subsidence prediction.

## Figures and Tables

**Table 1:** Date of Sentinel-1A ascending images.

Number	Date	Number	Date	Number	Date	Number	Date
1	20 May 2020	13	16 March 2021	25	29 December 2021	37	13 October 2022
2	13 June 2020	14	9 April 2021	26	22 January 2022	38	6 November 2022
3	7 July 2020	15	3 May 2021	27	15 February 2022	39	30 November 2022
4	12 August 2020	16	27 May 2021	28	11 March 2022	40	24 December 2022
5	5 September 2020	17	20 June 2021	29	4 April 2022	41	17 January 2023
6	29 September 2020	18	14 July 2021	30	28 April 2022	42	10 February 2023
7	23 October 2020	19	7 August 2021	31	22 May 2022	43	6 March 2023
8	16 November 2020	20	31 August 2021	32	15 June 2022	44	30 March 2023
9	10 December 2020	21	24 September 2021	33	9 July 2022	45	23 April 2023
10	3 January 2021	22	18 October 2021	34	2 August 2022	46	17 May 2023
11	27 January 2021	23	11 November 2021	35	26 August 2022		
12	20 February 2021	24	5 December 2021	36	19 September 2022		

**Table 2:** Date of Sentinel-1A descending images.

Number	Date	Number	Date	Number	Date	Number	Date
1	10 May 2020	13	22 February 2021	25	19 December 2021	37	8 November 2022
2	3 June 2020	14	18 March 2021	26	24 January 2022	38	2 December 2022
3	27 June 2020	15	11 April 2021	27	17 February 2022	39	7 January 2023
4	21 July 2020	16	5 May 2021	28	25 March 2022	40	31 January 2023
5	14 August 2020	17	10 June 2021	29	18 April 2022	41	24 February 2023
6	7 September 2020	18	4 July 2021	30	12 May 2022	42	20 March 2023
7	1 October 2020	19	28 July 2021	31	5 June 2022	43	13 April 2023
8	25 October 2020	20	21 August 2021	32	29 June 2022	44	7 May 2023
9	18 November 2020	21	14 September 2021	33	23 July 2022	45	31 May 2023
10	12 December 2020	22	8 October 2021	34	16 August 2022		
11	5 January 2021	23	1 November 2021	35	9 September 2022		
12	29 January 2021	24	25 November 2021	36	3 October 2022		

**Table 3:** BPNN Parameters.

Parameters	Value
Epochs	300
MES Goal	1 × 10^−6^
Learning Rate	0.1
Minimum Performance Gradient	1 × 10^−7^
Maximum Validation Failures	6
Display Frequency	25
Number of Input Neurons	5
Number of Sigmoid Hidden Layer Neurons	5
Number of Output Neurons	1
Input-to-Hidden Layer Activation Function	tansig
Hidden-to-Output Layer Activation Function	purelin
Learning Function	trainlm

**Table 4:** GA Parameters.

Parameters	Value
Population Sizes	50
Generations	200
Mutation Rate	0.1
Crossover Rate	0.8
Selection Function Parameters	0.09

**Table 5:** ABC Parameters.

Parameters	Value
Population Sizes	30
Maximum Iterations	100
Neighborhood Search Parameter	[−1, 1]
Limit Parameter	5

**Table 6:** Comparison of the prediction accuracy of models.

Point	BPNN		GA-BP		ABC-BP	
	MAE/mm	RMSE/mm	MAE/mm	RMSE/mm	MAE/mm	RMSE/mm
P1	4.07	4.40	2.93	3.16	1.57	1.77
P2	4.25	4.58	2.99	3.45	2.88	3.30
P3	4.31	4.61	2.23	2.37	1.61	1.89
P4	6.41	7.58	2.04	2.50	2.50	3.13
P5	6.36	7.14	2.88	3.20	2.36	3.09
P6	4.61	5.05	3.10	3.40	2.57	2.92
P7	5.88	6.76	2.84	2.53	2.92	3.64
P8	4.90	5.86	2.34	2.82	2.12	2.55

## Data Availability

The Sentinel-1A datasets can be acquired freely from the Copernicus and ESA: https://search.asf.alaska.edu/ (accessed on 7 October 2023). Rainfall data can be freely downloaded from the https://rp5.ru/ (accessed on 26 June 2024). GACOS data can be freely downloaded from the www.gacos.net (accessed on 31 October 2023).
